# The health behaviors differences among male and female school-age adolescents in the Middle East and North Africa region countries: a meta-analysis of the Global School-based Student Health Survey data

**DOI:** 10.3389/fpubh.2024.1448386

**Published:** 2024-08-26

**Authors:** Doha Achak, Asmaa Azizi, Abdelghaffar El-Ammari, Ibtissam Youlyouz Marfak, Elmadani Saad, Chakib Nejjari, Abderraouf Hilali, Karl Peltzer, Abdelghafour Marfak

**Affiliations:** ^1^Laboratory of Health Sciences and Technologies, Higher Institute of Health Sciences, Hassan First University of Settat, Settat, Morocco; ^2^Research, Development and Innovation Laboratory, Mundiapolis University, Casablanca, Morocco; ^3^National School of Public Health (Morocco), Rabat, Morocco; ^4^Euromed Research Center, Euromed University of Fes, Fes, Morocco; ^5^Faculty of Medicine, Pharmacy, and Dentistry, Sidi Mohamed Ben Abdellah University, Fes, Morocco; ^6^University of the Free State, Bloemfontein, South Africa; ^7^College of Medical and Health Science, Asia University, Taichung, Taiwan

**Keywords:** Global School-based Student Health Survey, adolescent and youth, healthy behaviors, Middle East and North Africa region, gender difference

## Abstract

**Background:**

Understanding gender disparities in adolescent health behaviors is crucial for developing targeted health promotion strategies. This study uses data from the Global School-based Student Health Survey (GSHS) across 17 MENA countries to analyze gender differences in adolescent health behaviors, aiming to provide a comprehensive overview for both boys and girls.

**Methods:**

This meta-analysis incorporates data from recent years of the GSHS, covering 17 MENA countries. The objective was to assess and compare health behaviors between adolescent girls and boys. A random-effects model was employed to calculate odds ratios for gender comparisons in these behaviors. Statistical analyses and modeling were performed using JAMOVI software.

**Results:**

In most MENA countries, boys consumed more vegetables compared to girls. Girls were less likely to frequent fast food establishments (OR = 0.82, 95% CI: 0.69–0.98) and generally exhibited better self-care behaviors. Boys reported a higher prevalence of physical altercations (OR = 2.18, 95% CI: 1.88–2.51) and were more involved in fights (OR = 3.00, 95% CI: 2.46–3.67). Girls were more likely to miss school without permission and were consistently described as kinder and more helpful across various income levels. However, in some countries such as Oman and Tunisia, boys missed school more frequently. There were no significant gender differences in parental oversight of homework or knowledge of students’ activities, but girls were reported to have stronger parental relationships and better parental understanding of their problems and free time, with exceptions in Morocco and the Palestinian Territory-Gaza. Boys were more likely to engage in smoking (OR = 3.57, 95% CI: 2.69–4.76) and other substance use. Conversely, girls reported higher levels of physical inactivity and loneliness, but also demonstrated greater kindness and helpfulness in school settings and stronger parental relationships.

**Conclusion:**

Policymakers in the MENA region should develop and implement gender-specific interventions targeting key areas such as hygiene practices, physical activity, and substance use. By focusing on these targeted strategies, they can address the distinct health behaviors and needs of both boys and girls. Effective interventions in these areas are crucial for improving overall health outcomes and promoting healthier lifestyles, thereby enhancing adolescent health and well-being across the region.

## Introduction

Adolescents aged 10–19 years old represent about 16% (1.3 billion) of the global population (1). Adolescence is a critical period in the transition to adulthood, characterized by significant behavioral, hormonal, and neurochemical changes (1). Adolescents are often seen as healthy, but this stage can bring about important risk factors for future health issues. These risks include physical inactivity, excessive sitting, tobacco and alcohol use, unhealthy eating habits, and inadequate hygiene. These behaviors can have lasting effects into adulthood (2).

The World Health Organization (WHO) recommends that adolescents participate in at least 60 min of moderate-to-vigorous-intensity Physical Activity (PA) daily to maintain a healthy lifestyle (3). However, numerous studies have pointed out that adolescents often do not meet this recommendation, indicating low levels of PA (4, 5). Additionally, sedentary behaviors, such as watching television, playing video games, or using computers—activities that require minimal energy expenditure—have been identified as detrimental to both mental and physical health during childhood (6–8). Moreover, tobacco and alcohol consumption is another concern that often begin during adolescence and can lead to negative health outcomes in adulthood (9). Studies have highlighted a significant association between tobacco use and injuries among students. Tobacco use can impair judgment and coordination amplifying the risk of accidents and injuries (10). Also, the association between physical fighting and tobacco can be explained by the fact that the consumption of psychoactive substances results in a stimulating effect that makes adolescents more aggressive and gets them more involved in situations of violence (11).

Health promotion programs implemented over the past two decades have aimed to reduce tobacco use, resulting in a decrease in smoking among middle and high school students globally. Unhealthy dietary habits, including fast food consumption, soda drinking, and low intake of fruits and vegetables, have been widely observed in adolescents (12, 13).

The World Health Organization (WHO), in collaboration with United Nations agencies and the Centers for Disease Control and Prevention (CDC), launched the Global School-based Student Health Survey (GSHS) in 2001 to monitor adolescent health behaviors and trends over time. This survey serves as a valuable tool for tracking a wide spectrum of adolescent health indicators, including tobacco, alcohol, and drug use, dietary habits, physical activity (PA), sedentary behaviors, mental health issues, violence, injuries, and hygiene practices (14). By gathering comprehensive data, the GSHS supports informed policy development and program implementation at regional, national, and local levels to promote adolescent health and well-being.

Conducted in over 120 countries worldwide (15), the GSHS has provided crucial insights into various health issues affecting adolescents. For instance, recent studies have highlighted that adolescents in Eswatini who experience bullying and food insecurity are at higher risk of suicidal behavior, with feelings of loneliness and anxiety potentially influencing this relationship (16). Moreover, adolescents exposed to physical violence often experience significant sleep disturbances, a trend observed consistently across different income groups and regions (17).

In the Middle East and North Africa (MENA) region, the GSHS has been conducted in several countries, including, among others, Tunisia, Iran, Lebanon, Morocco, Gaza and Egypt (18–23). It provided valuable data on the health and well-being of young people in this region. For example, GSHS data has highlighted high rates of tobacco use among adolescents in countries like Egypt (24), as well as high rates of physical inactivity and sedentary behavior across Arab countries (25). Data collected through the GSHS has been instrumental in informing policy and program development in the region. In Lebanon, for instance, the Ministry of Education utilized GSHS data to develop a school health policy that included the establishment of school health committees and the provision of health education materials to schools (26–32).

The study aims to achieve its objectives by utilizing data from the Global School-based Student Health Survey (GSHS) across 17 countries in the MENA region. Through meta-analysis, the study seeks to uncover and analyze gender variances in health behaviors among adolescents, providing comprehensive insights into both male and female behaviors.

The hypothesis of the study could be that gender differences in health behaviors among adolescents in the MENA region are influenced by a complex interplay of factors (27, 28). Specifically, it is expected that societal norms, cultural practices, economic status, and religious beliefs contribute significantly to the observed variations in health habits between boys and girls (29, 30).

By doing so, the study is meant to inform policymakers and stakeholders about the specific health needs and challenges faced by adolescents in the MENA region, thereby assisting in the development of targeted prevention and intervention strategies (31, 32). These strategies are intended to promote healthier behaviors and contribute to improving overall adolescent health and well-being in the diverse MENA context.

## Methods

### Data sources and outcomes

We conducted a meta-analysis using nationally representative GSHS datasets from the latest surveys conducted between 2007 and 2016 across 17 countries in the MENA region (Table 1). Each country’s most recent factsheet was included, resulting in a total of 17 datasets. The age groups targeted varied between countries, with some focusing on ages 13 to 15 years and others on ages 13 to 17 years for both males and females. The MENA region, spanning from Morocco to Iran, was the geographic scope of this study. The inclusion criteria required datasets to be nationally representative GSHS surveys, while datasets collected before 2007 or unpublished were excluded. The 17 countries included in this meta-analysis were: Algeria, Bahrain, Egypt, Iraq, Jordan, Kuwait, Lebanon, Libya, Mauritania, Morocco, Oman, Occupied Palestinian Territory Gaza, Qatar, Syrian Arab Republic, Tunisia, the United Arab Emirates, and Yemen.

**Table 1:** Correspondence of each question by country.

QN\Country	Morocco	Algeria	Bahrain	Iraq	UAE	Kuwait	Qatar	Libya	Tunisia	Mauritania	Yemen	SAR	OPTG	Lebanon	Oman	Jordan	Egypt
6	**+**	**+**	**+**	**+**	**+**	**+**	**+**	**+**	**+**	**+**	**+**	**+**	**+**	**+**	**+**	**+**	**+**
7	**+**	**+**	**+**	**+**	**+**	**+**	**+**	**+**	**+**	**+**	**+**	**+**	**+**	**+**	**+**	**+**	**+**
8	**+**	**+**	**+**	**+**	**+**	**+**	**+**	**+**	**+**	**+**	**+**	**+**	**+**	**+**	**+**	**+**	**+**
9	**+**	**+**	**+**	**+**	**+**	**+**	**+**	**−**	**−**	**+**	**+**	**+**	**+**	**+**	**+**	**−**	**+**
10	**+**	**+**	**+**	**+**	**+**	**+**	**+**	**_**	**_**	**+**	**+**	**+**	**+**	**+**	**+**	**_**	**+**
11	**+**	**+**	**+**	**+**	**+**	**+**	**+**	**−**	**+**	**+**	**+**	**+**	**+**	**+**	**+**	**+**	**+**
12	**+**	**+**	**+**	**+**	**+**	**+**	**+**	**+**	**+**	**+**	**+**	**+**	**+**	**+**	**+**	**+**	**+**
13	**+**	**+**	**+**	**+**	**+**	**+**	**+**	**+**	**+**	**+**	**+**	**+**	**+**	**+**	**+**	**+**	**+**
14	**+**	**+**	**+**	**+**	**+**	**+**	**+**	**+**	**+**	**+**	**+**	**+**	**+**	**+**	**+**	**+**	**+**
15	**+**	**+**	**+**	**+**	**+**	**+**	**+**	**+**	**+**	**+**	**+**	**−**	**+**	**+**	**+**	**+**	**+**
16	**+**	**+**	**+**	**+**	**+**	**+**	**+**	**+**	**+**	**+**	**+**	**−**	**+**	**+**	**+**	**+**	**+**
17	**+**	**+**	**+**	**+**	**+**	**+**	**+**	**+**	**+**	**+**	**+**	**+**	**+**	**+**	**+**	**+**	**+**
18	**−**	**+**	**+**	**+**	**+**	**+**	**+**	**+**	**+**	**+**	**+**	**−**	**+**	**+**	**+**	**+**	**+**
19	**+**	**+**	**+**	**+**	**+**	**+**	**+**	**−**	**−**	**+**	**+**	**−**	**+**	**+**	**+**	**+**	**+**
20	**+**	**+**	**+**	**+**	**+**	**+**	**+**	**+**	**+**	**+**	**+**	**−**	**+**	**+**	**+**	**+**	**+**
21	**+**	**+**	**+**	**+**	**+**	**+**	**+**	**−**	**+**	**+**	**+**	**−**	**+**	**+**	**+**	**+**	**+**
22	**+**	**−**	**+**	**+**	**+**	**+**	**+**	**−**	**+**	**+**	**+**	**+**	**+**	**+**	**+**	**+**	**+**
23	**+**	**−**	**+**	**+**	**+**	**+**	**+**	**−**	**+**	**+**	**+**	**+**	**+**	**+**	**+**	**+**	**−**
24	**+**	**−**	**+**	**+**	**+**	**+**	**−**	**−**	**+**	**+**	**+**	**−**	**+**	**+**	**+**	**+**	**−**
25	**+**	**−**	**+**	**+**	**+**	**+**	**−**	**−**	**+**	**+**	**+**	**−**	**+**	**+**	**−**	**+**	**−**
26	**+**	**−**	**+**	**+**	**+**	**+**	**−**	**−**	**+**	**−**	**+**	**−**	**+**	**+**	**−**	**−**	**−**
27	**+**	**−**	**+**	**+**	**+**	**+**	**+**	**−**	**+**	**+**	**+**	**+**	**+**	**+**	**+**	**+**	**+**
28	**+**	**+**	**+**	**+**	**+**	**+**	**+**	**+**	**+**	**+**	**+**	**+**	**+**	**+**	**+**	**+**	**+**
29	**+**	**+**	**+**	**+**	**+**	**+**	**+**	**+**	**+**	**+**	**+**	**+**	**+**	**+**	**+**	**+**	**+**
30	**+**	**+**	**+**	**+**	**+**	**+**	**+**	**+**	**+**	**+**	**+**	**+**	**+**	**+**	**+**	**+**	**+**
31	**+**	**+**	**+**	**+**	**+**	**+**	**+**	**+**	**+**	**+**	**+**	**+**	**+**	**+**	**+**	**+**	**+**
32	**+**	**+**	**+**	**+**	**+**	**+**	**+**	**+**	**+**	**+**	**+**	**+**	**+**	**+**	**+**	**+**	**+**
33	**+**	**+**	**+**	**+**	**+**	**+**	**+**	**+**	**+**	**+**	**+**	**+**	**+**	**+**	**+**	**+**	**+**
34	**−**	**−**	**−**	**−**	**−**	**−**	**−**	**−**	**−**	**−**	**−**	**+**	**−**	**+**	**−**	**−**	**−**
35	**−**	**−**	**−**	**−**	**−**	**−**	**−**	**−**	**−**	**−**	**−**	**+**	**−**	**+**	**−**	**−**	**−**
36	**−**	**−**	**−**	**−**	**−**	**−**	**−**	**−**	**−**	**−**	**−**	**+**	**−**	**+**	**−**	**−**	**−**
37	**−**	**−**	**−**	**−**	**−**	**−**	**−**	**−**	**−**	**−**	**−**	**−**	**−**	**+**	**−**	**−**	**−**
38	**−**	**−**	**−**	**−**	**−**	**−**	**−**	**−**	**−**	**−**	**−**	**−**	**−**	**+**	**−**	**−**	**−**
39	**−**	**−**	**−**	**−**	**−**	**−**	**−**	**−**	**−**	**−**	**−**	**−**	**−**	**+**	**−**	**−**	**−**
40	**+**	**+**	**+**	**+**	**−**	**−**	**−**	**−**	**+**	**−**	**−**	**−**	**+**	**+**	**+**	**−**	**−**
41	**+**	**+**	**+**	**+**	**−**	**−**	**−**	**−**	**−**	**+**	**−**	**−**	**+**	**+**	**+**	**−**	**−**
42	**+**	**+**	**+**	**+**	**−**	**−**	**−**	**−**	**−**	**+**	**−**	**−**	**+**	**+**	**+**	**−**	**−**
43	**+**	**+**	**+**	**+**	**−**	**−**	**−**	**−**	**−**	**+**	**−**	**−**	**+**	**+**	**+**	**−**	**−**
44	**−**	**−**	**−**	**−**	**+**	**−**	**−**	**−**	**−**	**+**	**−**	**−**	**−**	**−**	**−**	**−**	**−**
45	**−**	**−**	**−**	**−**	**−**	**−**	**−**	**−**	**−**	**+**	**−**	**−**	**−**	**−**	**−**	**−**	**−**
46	**−**	**−**	**−**	**−**	**−**	**−**	**−**	**−**	**−**	**+**	**−**	**−**	**−**	**−**	**−**	**−**	**−**
47	**−**	**−**	**−**	**−**	**−**	**−**	**−**	**−**	**−**	**+**	**−**	**−**	**−**	**−**	**−**	**−**	**−**
48	**−**	**−**	**−**	**−**	**−**	**−**	**−**	**−**	**−**	**+**	**−**	**−**	**−**	**−**	**−**	**−**	**−**
49	**+**	**+**	**+**	**+**	**+**	**+**	**+**	**+**	**+**	**+**	**+**	**+**	**+**	**+**	**+**	**+**	**+**
50	**+**	**+**	**+**	**+**	**+**	**+**	**+**	**+**	**+**	**−**	**+**	**+**	**+**	**+**	**+**	**+**	**+**
51	**+**	**+**	**+**	**+**	**+**	**+**	**+**	**−**	**−**	**+**	**+**	**+**	**+**	**+**	**+**	**−**	**+**
52	**+**	**+**	**+**	**+**	**+**	**+**	**+**	**+**	**+**	**+**	**+**	**+**	**+**	**+**	**+**	**+**	**+**
53	**+**	**−**	**+**	**+**	**+**	**+**	**+**	**+**	**+**	**+**	**+**	**+**	**+**	**+**	**+**	**+**	**+**
54	**+**	**−**	**+**	**+**	**+**	**+**	**+**	**+**	**+**	**+**	**+**	**+**	**+**	**+**	**+**	**+**	**+**
55	**+**	**−**	**+**	**+**	**+**	**+**	**+**	**+**	**+**	**+**	**+**	**+**	**+**	**+**	**+**	**+**	**+**
56	**+**	**−**	**+**	**+**	**+**	**+**	**+**	**+**	**+**	**+**	**+**	**+**	**+**	**+**	**+**	**+**	**+**
57	**+**	**−**	**+**	**+**	**+**	**+**	**+**	**+**	**+**	**+**	**+**	**+**	**+**	**+**	**+**	**+**	**+**
58	**+**	**−**	**+**	**+**	**+**	**+**	**+**	**−**	**−**	**+**	**+**	**+**	**+**	**+**	**+**	**−**	**+**

−, Country missing QN; +, Country using QN.

The meta-analysis covered various health behaviors, such as dietary habits (including skipping meals and consumption of fruits and vegetables), physical activity, and sedentary behavior. It also examined tobacco and substance use, instances of injury and violence (including recent injuries, bullying experiences, involvement in fights, and physical attacks within the past year), and hand hygiene practices (like washing hands before meals, after using the restroom, and using soap).

**Figure 1:**
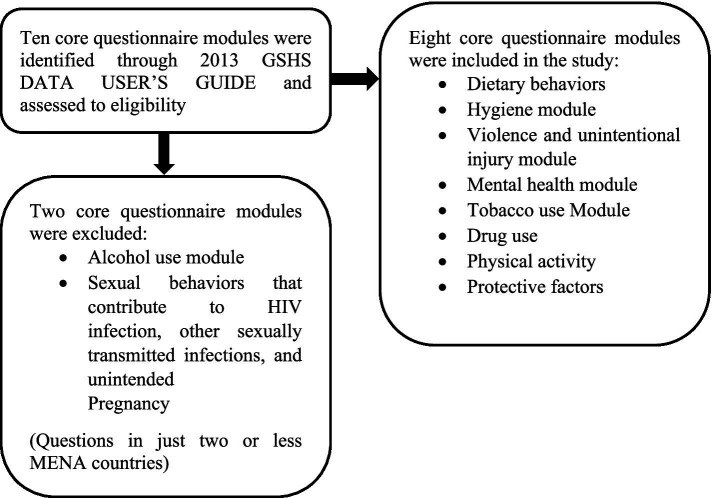
Flow diagram for the modules retrieved from the Global School-based Student Health Survey (GSHS) and included in the present study.

The study also investigated psychological health indicators such as social relationships (having friends and feelings of loneliness), sleep quality, and suicidal tendencies. Protective factors considered in the analysis included school attendance, parental support, and peer relationships (see Figure 1).

**Figure 2:**
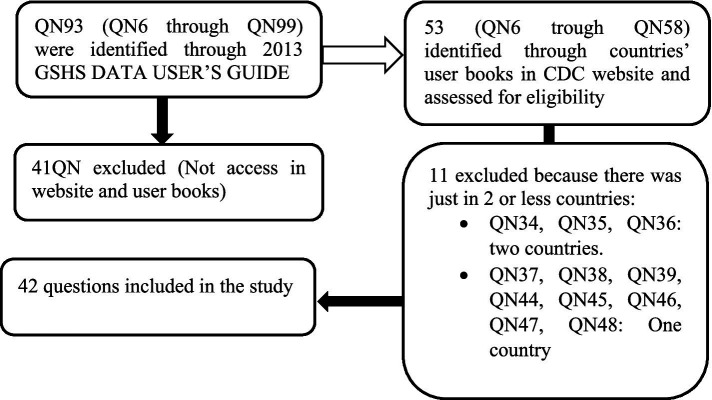
Flow diagram for variables of the Global School-based Student Health Survey (GSHS) which were included in the study. QN: Standard variable name (dichotomic variable) across all GSHS data file.

In the codebooks of the GSHS data, variables were symbolized by QN, which stands for converted questions in dichotomized variables. Dichotomized variables divide students into two groups: those who report a particular behavior or knowledge and those who do not. These variables are created by combining responses from the original question into the response of interest, which is the typical way of reporting variables. For example, QN6 is the dichotomous variable that corresponds to question Q6, which has been dichotomized to indicate (students who went hunger to school most of the time or always because of insufficient food in their home during the 30 days before the survey) (Supplementary File 1). Dichotomous variables are created during data processing and are consistent across all GSHS data files, allowing for comparable analyses across countries. In this study, 42 variables were included, from QN6 to QN58 (Figure 2).

### Ethical statement

This is a meta- analysis of 17 GSHS countries data obtained from the World Health Organization (WHO) (33). The study was approved by the local committee of institutional review board of Hassan First University according to the Moroccan law 28\13-art.2 in accordance with the relevant guidelines and requirements of the Helsinki declaration of ethics.

### Statistical analysis

In this meta-analysis, we analyzed all questions from QN6 to QN58, except QN34, QN35, QN36, QN37, QN38, QN39, QN44, QN45, QN46, QN47, and QN48. These questions were excluded because they were answered by students in two or fewer countries within the MENA region. To compare the girls’ and the boys’ behaviors, we estimated the odds ratios of all questions considering females as the reference category. For example, from QN6: ‘Percentage of students who most of the time or always went hungry to school’, if odds-ratio > 1 then boys went hungry more than girls. The analysis included 61,531 school-age adolescents aged between 13 and 17 years old from surveys conducted in the 17 MENA region countries (Table 2).

**Table 2:** Year of survey, number, and age range of students participated in Global School-based student health survey (GSHS) for the 17 countries considered in the present study.

Country	Survey year	Number of students	Age (in years)
Algeria	2011	4,532	13–15
Bahrain	2016	7,141	13–17
Egypt	2011	2,568	13–15
Iraq	2012	2038	13–15
Jordan	2007	2,197	13–15
Kuwait	2015	3,637	13–17
Lebanon	2017	5,708	13–17
Libya	2007	2,242	13–15
Morocco	2016	6,745	13–17
Qatar	2011	2021	13–15
Occupied Palestinian Territory GAZA	2010	2,677	13–15
Oman	2015	3,486	13–17
Syrian Arab Republic	2010	3,102	13–15
Yemen	2014	2,655	13–17
United Arab Emirates	2016	5,849	13–17
Mauritania	2010	2063	13–15
Tunisia	2008	2,870	13–15

The meta-analyses were performed using the JAMOVI software version 2.3.28 via the ‘MAJOR’ package. The analysis utilized the log odds ratios as the outcome measure, which was subsequently converted to odds ratios. We employed a random-effects model to fit the data. The amount of heterogeneity (tau^2^) was estimated using the restricted maximum-likelihood estimator (20). In addition to the estimate of tau^2^, we reported the Q-test for heterogeneity, and the I^2^ statistic. If any heterogeneity was detected, a prediction interval for the true outcomes was provided. To check for funnel plot asymmetry, we conducted the rank correlation test and the regression test using the standard error of the observed outcomes as a predictor.

## Results

### Dietary behaviors

Regarding hunger and fruit/vegetable consumption, in Kuwait and the United Arab Emirates, boys went to school less hunger than girls, while in Oman (OR = 1.65), Morocco (OR = 1.32), and Libya (OR = 1.30), boys experienced more hunger than girls. There were no significant differences between males and females in this regard in the other countries.

Moreover, there were no significant differences between boys and girls in terms of consuming fruits two or more times per day in any of the considered countries. However, boys tended to consume vegetables more frequently than girls (OR = 1.13, 1.04–1.21) in all countries, regardless of income, with particularly high odds ratios observed in Bahrain (OR = 1.6) and Tunisia (OR = 1.31). The income of the countries did not affect these behaviors.

Regarding carbonated soft drink consumption, 13 countries were included in the analysis, and no significant differences were observed between boys and girls about this. Fourteen countries were included in the analysis of fast-food consumption, boys were found to consume fast food more frequently than girls in these countries, except for Qatar and Mauritania.

### Oral health and hand washing

Overall, girls reported brushing their teeth one or more times per day more frequently than boys in 16 countries. Besides, among 17 countries, girls washed their hands before eating more frequently than boys in the Syrian Arab Republic, Algeria, Qatar, Libya, Oman, Jordan, Morocco, and Iraq. The largest difference was observed in Iraq (OR = 13.6), while no significant differences were found between males and females in the other countries. Moreover, except for Mauritania, Tunisia, and Algeria, where there were no significant gender differences in terms of handwashing after using toilets or latrines, boys washed their hands less frequently than girls in all 14 countries. The largest odds ratio was observed in Morocco (OR = 3.0). Girls were also found to use soap more frequently than boys when washing their hands, except for Mauritania (Table 3).

**Table 3:** Result of meta-analysis on dichotomized questions QN for the GSHS survey comparing males and females.

Item	Description	Odds ratio [95% CI]	I^2^	H^2^	Tau^2^	Q	pQ	Comments
QN6	Percentage of students who most of the time or always went hungry (because there was not enough food in their home during the 30 days before the survey).	1.11 [1.00, 1.22]	64.7	2.8	0.027	47.5	<0.001	Significant difference
QN7	Percentage of students who did eat fruit 2 or more times per day (during the 30 days before the survey).	0.97 [0.87, 1.09]	83.3	6.0	0.044	97.0	1.24E-13	No significant difference
QN8	Percentage of students who did eat vegetables 3 or more times per day (during the 30 days before the survey).	1.13 [1.04, 1.21]	54.1	2.2	0.012	35.9	0.003	Significant difference
QN9	Percentage of students who did not drink carbonated soft drinks (excluding diet soft drinks, during the 30 days before the survey).	0.97 [0.65, 1.43]	98.96	95.71	0.52	1251.0	<0.001	No significant difference
QN10	Percentage of students who did not eat food from a fast-food restaurant (during the 7 days before the survey).	0.82 [0.69, 0.98]	94.21	17.27	0.101	192.05	<0.001	Significant difference
QN11	Percentage of students who usually cleaned or brushed their teeth (one or more times per day during the 30 days before the survey).	0.47 [0.41, 0.56]	91.91	12.36	0.088	182.12	<0.001	significant difference
QN12	Percentage of students who never or rarely washed their hands before eating (during the 30 days before the survey).	1.58 [1.19, 2.12]	95.43	21.90	0.35	298.89	<0.001	Significant difference
QN13	Percentage of students who never or rarely washed their hands after using the toilet or latrine (during the 30 days before the survey).	1.71 [1.51, 1.95]	71.11	3.46	0.05	62.35	<0.001	Significant difference
QN14	Percentage of students who never or rarely used soap when washing their hands (during the 30 days before the survey).	1.84 [1.63, 2.05]	67.83	3.02	0.04	49.72	<0.001	Significant difference
QN15	Percentage of students who were physically attacked (one or more times during the 12 months before the survey).	2.18 [1.88, 2.51]	92.97	13.84	0.08	179.79	<0.001	Significant difference
QN16	Percentage of students who were in a physical fight (one or more times during the 12 months before the survey).	3.00 [2.46, 3.67]	96.68	30.15	0.16	355.17	<0.001	Significant difference
QN17	Percentage of students who were seriously injured (one or more times during the 12 months before the survey).	1.95 [1.79,2.16]	85.56	6.93	0.034	93.09	<0.001	Significant difference
QN18	Percentage of students who reported that their most serious injury was a broken bone or dislocated joint (among students who were seriously injured during the 12 months before the survey).	1.56 [1.35, 1.80]	65.2	2.87	0.005	39.28	<0.001	Significant difference
QN19	Percentage of students who reported that their most serious injury was caused by a motor vehicle accident or being hit by a motor vehicle (among students who were seriously injured during the 12 months before the survey).	1.92 [1.57, 2.34]	62.18	2.64	0.084	32.39	0.001	Significant difference
QN20	Percentage of students who were bullied (on one or more days during the 30 days before the survey).	1.42 [1.26, 1.60]	90.6	10.640	0.055	182.03	<0.001	Significant difference
QN21	Percentage of students who were bullied most often by being hit, kicked, pushed, shoved around, or locked indoors (among students who were bullied during the 30 days before the survey).	1.95 [1.67, 2.27]	61.46	2.595	0.055	34.99	0.001	Significant difference
QN22	Percentage of students who most of the time or always felt lonely (during the 12 months before the survey).	0.66 [0.59, 0.75]	82.79	5.809	0.044	72.08	<0.001	Significant difference
QN23	Percentage of students who most of the time or always were so worried about something that they could not sleep at night (during the 12 months before the survey).	0.57 [0.52, 0.64]	77.65	4.475	0.031	52.50	<0.001	Significant difference
QN24	Percentage of students who seriously considered attempting suicide (during the 12 months before the survey).	0.88 [0.79,0.98]	76.14	4.129	0.026	43.90	<0.001	Significant difference
QN25	Percentage of students who made a plan about how they would attempt suicide (during the 12 months before the survey).	0.84 [0.77,0.92]	45.84	1.85	0.008	18.97	0.041	Significant difference
QN26	Percentage of students who attempted suicide (one or more times during the 12 months before the survey).	0.96 [0.88,1.05]	48.57	1.944	0.009	15.17	0.047	No significant difference
QN27	Percentage of students who did not have any close friends.	0.97 [0.84,1.12]	76.16	4.194	0.238	53.44	<0.001	No significant difference
QN28	Percentage of students who tried a cigarette before age 14 years (for the first time among students who ever smoked cigarettes).	1.24 [0.97,1.60]	81.8	5.5	0.199	79.4	<0.001	No significant difference
QN29	Percentage of students who currently smoked cigarettes (on at least 1 day during the 30 days before the survey).	3.57 [2.68,4.75]	95.4	21.6	0.338	259.9	<0.001	Significant difference
QN30	Percentage of students who use any tobacco products other than cigarettes, such as Shisha, Tabac a snifer, Kala? (on at least 1 day during the 30 days before the survey).	2.72 [2.11,3.53]	95.68	23.14	0.265	329.31	<0.001	Significant difference
QN31	Percentage of students who tried to stop smoking cigarettes, during the past 12 months?	1.45 [1.22,1.72]	38.23	1.62	0.047	28.258884	0.029	Significant difference
QN32	on how many days have people smoked in your presence (on at least 1 day during the 7 days before the survey).	1.37 [1.19,1.57]	93.89	16.36	0.078	284.95	<0.001	Significant difference
QN33	Which of your parents or guardians use any form of tobacco?	1.02 [0.93,1.13]	84.31	6.37	0.033	87.94	<0.001	No significant difference
QN40	Percentage of students who used drugs before age 14 years (for the first time among students who ever used drugs).	0.96 [0.61,1.52]	72.23	3.601	0.316	33.78	<0.001	No significant difference
QN41	Percentage of students who ever used marijuana (one or more times during their life).	4.26 [2.36,7.69]	92.77	13.824	0,6,433	78.73	<0.001	Significant difference
QN42	Percentage of students who currently used marijuana (one or more times during the 30 days before the survey).	4.14 [2.44,7.03]	90.4	10.414	0.515	70.68	<0.001	Significant difference
QN43	Percentage of students who ever used amphetamines or methamphetamines (one or more times during their life).	3.49 [1.80,6.69]	93.84	16.23	0.813	98.73	<0.001	Significant difference
QN49	Percentage of students who were not physically active (for at least 60 min per day on any day during the 7 days before the survey).	0.60 [0.48,0.75]	96.66	29.91	0.203	371.35	<0.001	Significant difference
QN50	Percentage of students who did not walk or ride a bicycle to or from school (during the 7 days before the survey)	0.74 [0.63,0.86]	94.74	19.01	0.100	290.70	<0.001	Significant difference
QN51	Percentage of students who did not attend physical education classes (each week during this school year).	0.79 [0.64,0.97]	96.18	26.20	0.16	342.72	<0.001	Significant difference
QN52	Percentage of students who spent three or more hours per day doing sitting activities (sitting and watching television, playing computer games, talking with friends when not in school or doing homework during a typical or usual day)	0.95 [0.79,1.13]	96.04	25.27	0.132	398.02	<0.001	No significant difference
QN53	Percentage of students who missed classes or school without permission (on one or more days during the 30 days before the survey).	1.25 [1.07,1.43]	93.57	15.56	0.08	267.32	<0.001	Significant difference
QN54	Percentage of students who reported that most of the students in their school were most of the time or always kind and helpful (during the 30 days before the survey).	0.66 [0.61,0.73]	81.15	5.304	0.02	75.03	<0.001	Significant difference
QN55	Percentage of students who reported that their parents or guardians most of the time or always checked to see if their homework was done (during the 30 days before the survey).	1.07 [0.96,1.20]	89.62	9.63	0.044	181.50	<0.001	No significant difference
QN56	Percentage of students who reported that their parents or guardians most of the time or always understood their problems and worries (during the 30 days before the survey).	0.88 [0.81,0.95]	73.91	3.833	0.015	57.75	<0.001	Significant difference
QN57	Percentage of students who reported that their parents or guardians most of the time or always really knew what they were doing with their free time (during the 30 days before the survey).	0.79 [0.72,0.88]	87.07	7.74	0.035	119.91	<0.001	Significant difference
QN58	Percentage of students who reported that their parents or guardians never or rarely went through their things without their approval (during the 30 days before the survey).	0.79 [0.70,0.90]	85.95	7.118	0.042	75.57	<0.001	Significant difference

### Physical attacks, physical fights, and injuries

Boys were physically attacked twice as often as girls, with Tunisia, Libya, and Jordan showing particularly high odds ratios of 3.78, 3.53, and 3.16, respectively. High-income countries showed similar patterns, except for Oman, where boys were physically attacked less frequently than girls in other income categories.

Overall, boys were involved in physical fights three times more often than girls (OR = 3.00, 2.46–3.67), with Tunisia showing a particularly high odds ratio of 7.0. In Iraq, no significant difference was found between male and female students.

Regarding injuries, boys were seriously injured twice as often as girls in all countries included in the analysis, with Egyptian students showing the highest odds ratio of 3.19. The percentage of students reporting broken bones or dislocated joints as their most serious injury was twice as high in boys compared to girls in 8 out of the 15 countries studied, while in Iraq, the difference was approximately three times higher. It should be mentioned that there are no gender differences about this in all countries that are classified as lower-middle-income countries (34).

- Low-income countries: Yemen
- Lower middle-income countries: Algeria, Egypt, Morocco, Mauritania, Jordan, Syria, Tunisia
- Upper middle-income countries: Iraq, Lebanon, Palestine, Libya
- High-income countries: Bahrein, Kuwait, Qatar, Oman, Emirates Arab Unis

However, in eight countries (Lebanon, Qatar, Oman, Algeria, Yemen, Morocco, United Arab Emirates, and the Palestinian Territory-Gaza), boys reported experiencing motor vehicle accidents or being hit by a motor vehicle as their most serious injury twice as often as girls.

### Bulling behaviors

Across most countries, boys reported experiencing higher levels of bullying compared to girls, especially in Yemen, a low-income country, and Lebanon, with odds ratios of 1.88 and 1.92, respectively. The only exception was Algeria, where girls reported higher levels of bullying than boys. In Oman, Egypt, and Mauritania, there were no significant gender differences in bullying. Boys were more likely than girls to report being frequently bullied through physical acts such as hitting, kicking, pushing, shoving, or being locked indoors in all countries, except for Mauritania, Yemen, Qatar, and Kuwait (Table 3).

### Psychological and mental health

Regarding feelings of loneliness, girls consistently reported feeling lonely more often than boys in all countries except Qatar, Oman, Mauritania, and the Palestinian Territory-Gaza. Also, girls expressed higher levels of worry, causing them to lose sleep at night, compared to boys in all countries except Qatar and Mauritania. In high-income countries, the situation was similar, except for Qatar, where the same pattern as low-income countries persisted.

In the majority of countries, females were observed to seriously considered attempting suicide, except in Yemen where boys reported higher rates. In Iraq, Jordan, Oman, Mauritania, and the Palestinian Territory-Gaza, no significant gender differences were found in this regard. Regarding making a plan for attempting suicide, except for Mauritania, Morocco, Jordan, Kuwait, Yemen, Tunisia, and the Palestinian Territory-Gaza, girls reported making plans more frequently than boys in the remaining 10 countries. Income level did not affect suicide behaviors. No significant gender differences were observed for students who had attempted suicide, lacked close friends, or tried smoking cigarettes before the age of 14.

### Tobacco and drug use

Boys were found to currently smoke cigarettes at least once during the 30 days preceding the survey four times more often than girls, with an overall odds ratio of (3.57, 2.68–4.75) (Table 3). The largest difference was observed in lower middle-income countries such as Algeria, where boys were 14 times more likely to smoke than girls, followed by Egypt (OR = 5.75) and Tunisia (OR = 5.10) (Supplementary File 2). No significant gender differences were found in Mauritania and Yemen. In high and upper middle-income countries, the percentages of smoking students were similar and relatively low. Boys were three times more likely than girls to use other tobacco products such as shisha, snuff, or Kala. The largest differences were observed in Algerian, Omani, and Tunisian students, with odds ratios of 9.13, 5.58, and 4.09, respectively. Income level did not have an impact in this case. Significant differences were observed in upper middle-income countries, where boys were more likely to attempt to quit smoking compared to girls, with odds ratios of 1.70, 1.51, 5.42, and 4.66 for Jordan, Lebanon, Iraq, and Libya, respectively. However, no significant gender differences were found in Jordan, Libya, Syria, Mauritania, and Tunisia in terms of exposure to smoking in their presence. In other countries, boys reported exposure to smoking at approximately twice the rate of girls, except for Lebanon, where the pattern was reversed. No significant gender differences were found regarding parents or guardians who used any form of tobacco, or among students who started using drugs before the age of 14. In the majority of countries, boys reported higher rates of marijuana use (one or more times during their lifetime) compared to girls, with the largest difference observed in Algeria (OR = 20.70). However, no gender differences were found in Mauritania and Iraq. For the two higher-income countries included in the analysis, Oman and Bahrain, the prevalence rates were similar for boys and girls. Regarding students who currently used marijuana (one or more times during the 30 days before the survey) and amphetamines or methamphetamines, boys reported using marijuana about four times more often than girls in all countries studied, except for Mauritania and Iraq where no significant difference was found between genders.

### Physical activity and sedentary behaviors

Regarding physical activity (Table 3), girls were found to be more physically inactive than boys in all countries, except for Iraq where girls were more physically active than boys (Supplementary File 2). Analyzing students who did not walk or ride a bicycle to or from school in 17 countries, it was observed that boys walked or rode bicycles more often than girls in the majority of countries, except for the Syrian Arab Republic, Algeria, Libya, Tunisia, Qatar, Palestinian Territory GAZA where no significant difference was found between males and females. In Egypt, girls walked or rode bicycles more often than boys.

Girls who did not attend physical education classes were more prevalent than boys in the majority of countries, except for some high-income countries (Qatar, Oman, Bahrain, United Arab Emirates) and Morocco, where no significant gender difference was found. In Libya, boys were 1.43 times more likely to not attend physical education classes than girls. Regarding students who spent three or more hours per day engaging in sitting activities, no significant difference was found between genders (OR = 0.95, 0.79–1.13).

### Experiences at school and at home

In Kuwait and the United Arab Emirates, girls were more likely to miss classes or school without permission (on one or more days during the 30 days before the survey) compared to boys. Conversely, in Oman, Tunisia, Libya, Lebanon, Morocco, and Egypt, boys were more likely to miss classes or school without permission than girls. No significant gender difference was observed in other countries. Likewise, girls were more often described as kind and helpful compared to boys in all high-income, upper middle-income, lower middle-income, and low-income countries, except for Mauritania where gender did not affect this dimension (Supplementary File 2).

No significant difference was found between male and female students in reporting whether their parents or guardians checked to see if their homework was done most of the time or always in all 16 countries studied. In Oman, Morocco, Qatar, the Palestinian Territory-Gaza, the United Arab Emirates, and the Syrian Arab Republic, girls reported that their parents or guardians understood their problems and worries most of the time or always (during the 30 days before the survey) one time more often than boys, regardless of the countries’ income category. Regarding the percentage of students who reported that their parents or guardians knew what they were doing with their free time most of the time or always (during the 30 days before the survey), girls were one time more likely than boys in 11 countries, except for the Palestinian Territory-Gaza, Jordan, Kuwait, Iraq, and Yemen where no significant difference was found between males and females. Similarly, the same pattern was observed for the percentage of students who reported that their parents or guardians never or rarely went through their things without their approval (during the 30 days before the survey). Girls were more likely than boys in all countries, except for Morocco, Kuwait, and the Palestinian Territory-Gaza, where no significant gender difference was observed (Supplementary File 2).

## Discussion

This research sought to explore gender disparities in health behaviors among adolescent students using data from the GSHS across 17 countries in the Middle East and North Africa. The meta-analyses yielded diverse findings based on students’ gender, summarized in a students’-sex-behavior map (Supplementary File 2).

Regarding dietary behaviors, boys in Bahrain, Qatar, Tunisia, and the Occupied Palestinian Territory-Gaza reported consuming more vegetables than girls, while no significant gender differences were observed in other MENA countries. These findings align with international research (35, 36). One possible explanation for these discrepancies is differing food preferences between genders (37). Bere et al. suggest that the availability of vegetables at home may influence these variations (38). Conversely, Rasmussen et al. found that girls generally have a higher intake of fruits and vegetables (36), implying that boys may prefer energy-dense foods to satisfy their higher energy needs (39). Historical gender roles, where men traditionally hunted and women gathered, might also contribute to these dietary patterns (40).

A notable observation is that the tendency for boys to consume more vegetables in specific countries could be indicative of a broader cultural or societal influence on dietary habits. For example, in regions where boys are encouraged to consume more nutrient-dense foods to support physical growth or strength, such as in athletic or labor-intensive roles, this pattern might emerge. Furthermore, the consistency of girls’ higher fruit and vegetable intake in many studies could reflect a stronger societal emphasis on these foods for females, potentially linked to perceptions of health and wellness (37, 39). Addressing these gender-specific dietary patterns could lead to more targeted and effective nutritional interventions, ensuring that both boys and girls receive balanced and healthful diets.

Furthermore, the rising consumption of fast food can be attributed to its rapid service, convenience, appealing taste, and affordable prices (37, 41). In MENA countries, boys generally consume fast food more frequently than girls, except in Qatar and Mauritania, where this trend is reversed (42). This difference may stem from the time constraints commonly experienced by males and the prevalent availability of fast food in school settings (43, 44). The increased frequency of fast food consumption among boys likely reflects distinct gender-related lifestyle and dietary patterns. Boys may prioritize convenience and immediate gratification, potentially due to greater energy needs or less time available for meal preparation. In contrast, girls may exhibit more selective eating habits, potentially influenced by health consciousness or specific dietary guidelines aimed at females. Additionally, cultural factors, including those associated with the Mediterranean diet, affect food preferences and accessibility, contributing to regional differences in fast food consumption between genders. Addressing these disparities through targeted educational and policy measures could help balance dietary habits and promote healthier eating patterns for both genders.

Investigating hygiene practices as preventive measures against illness and unhealthy behaviors in adolescents is crucial, given their significant impact on community health by curbing infection spread: Effective hand washing is essential in protecting against various infections, from common colds to more severe diseases (45, 46). The study indicates that girls consistently exhibit superior hand hygiene practices compared to boys across all MENA countries examined (47). Additionally, girls report more frequent tooth brushing and soap usage than their male counterparts. This discrepancy highlights the influential role of parental guidance in shaping adolescents’ hygiene behaviors, as girls often receive more supervision and encouragement regarding cleanliness. Consequently, it is not surprising that girls are more likely to adopt and maintain these hygiene practices diligently (48). There may be a greater emphasis on hygiene education for girls, driven by societal expectations and health campaigns that target female adolescents more intensively. This educational focus can result in more frequent and diligent hygiene practices among girls. Also, boys might prioritize other activities over hygiene, or they might not perceive the same level of urgency regarding hygiene practices (47). This can be influenced by differing behavioral patterns and attitudes toward health and cleanliness. These results stress the crucial role of parental involvement in promoting healthy habits, which enhances overall community health and well-being.

Bullying behavior manifests as either physical aggression or verbal abuse, with boys typically engaging in physical intimidation or threats regardless of the victim’s gender, whereas girls primarily use verbal bullying, often targeting other girls (49). The study found that boys experienced higher rates of bullying compared to girls across all MENA countries, which aligns with numerous prior studies (50, 51). However, this pattern was not observed in Algeria. This exception highlights how societal norms influence bullying behaviors, where masculinity is often associated with physical aggression and femininity with verbal aggression. In Algeria, this trend has been corroborated by earlier research (52). Cultural norms governing gender interactions may explain the variation in bullying experiences. Specifically, in societies where physical aggression is more socially accepted for boys, higher rates of physical bullying are observed, while girls may resort to verbal forms of aggression, reflecting different societal expectations and norms (53, 54). These findings illustrate the significant impact of gender roles and cultural contexts on bullying behaviors throughout the MENA region. Overall, the results reflect a complex interplay of gender socialization, cultural norms, and regional differences, underscoring the need for culturally sensitive interventions and educational reforms to address bullying effectively across different contexts.

Regarding suicidal behaviors, girls were reported to have a higher prevalence of serious suicide attempts and suicide planning compared to boys in the majority of countries in the MENA region. Gender differences in suicidal behavior may be attributed to variations in emotional and behavioral problems (55, 56). In the MENA region, girls may be more prone to internalizing their emotions, leading to a higher susceptibility to depression and anxiety (57), which are associated with suicidal behaviors: Furthermore, the limited awareness of mental health issues and the stigma associated with psychiatric conditions in many Arab countries contribute to a scarcity of mental health services, which can exacerbate suicidal tendencies (58). These results emphasize the urgent requirement for tailored interventions that address gender-specific vulnerabilities to suicidal behaviors in the MENA region. The elevated occurrence of serious suicide attempts among girls underscores the significance of promoting emotional and psychological well-being with culturally appropriate strategies. By targeting factors like depression and anxiety, we can cultivate environments that enhance mental health resilience and decrease the occurrence of suicidal behaviors among youth.

Concerning Tobacco and drug use, the findings revealed that boys in the majority of Eastern Mediterranean region countries have higher rates of tobacco use compared to girls. Boys might have greater social acceptance or encouragement for engaging in tobacco and drug use compared to girls, who may face stronger social disapproval. Additionally, boys often have more independence and less supervision than girls, which can lead to increased exposure to environments where tobacco and drug use are prevalent. Additionally, peer pressure among boys may further contribute to higher rates of substance use. This trend aligns with Ma et al.’s study, which noted that the Eastern Mediterranean region is one of only two global regions where cigarette use has not declined over a decade (59). This stagnation in reducing cigarette use may disproportionately affect boys due to their higher engagement with tobacco. Furthermore, the overall prevalence of injuries was higher among boys than girls, with falls being the main cause (60). In Egypt, which was the most affected country in this study in terms of injuries, Wahdan MM et al. found a significant association between smoking and injuries (10). These factors collectively explain the observed differences in tobacco and drug use between boys and girls and emphasize the need for targeted public health interventions to address these disparities and improve health outcomes in the region.

In terms of physical activity, the study consistent with other research in the Arab region and globally, reveals a notable gender disparity in physical activity, with girls being less physically active than boys (36, 61, 62). Several factors contribute to this difference. First, girls are less likely to participate in organized sports (63), which limits their overall physical activity levels. Girls may experience body image concerns or lower self-esteem related to physical appearance, which can negatively impact their willingness to engage in physical activities. Second, they often receive less social and environmental support for engaging in physical activities compared to boys (64). In many cultures, traditional gender roles may discourage girls from participating in physical activities. Activities deemed appropriate for boys might be considered less suitable for girls, leading to reduced participation, also, girls may prioritize academic or other extracurricular activities over physical activities, leading to lower levels of engagement in sports and exercise. Finally, girls may find less enjoyment in physical education, which can further discourage participation. Limited visibility of female athletes or active female role models can reduce girls’ motivation to participate in sports and physical activities, also Concerns about safety or social judgment can deter girls from engaging in physical activities, especially in environments where they feel uncomfortable or unwelcome. These factors collectively account for the observed gender gap in physical activity among youth (65).

Regarding experiences at school and home, the findings reveal notable gender differences. Girls are generally more helpful in the classroom, demonstrating a calm demeanor and contributing to a harmonious learning environment. They also tend to have stronger relationships with their parents and higher levels of confidence. Research indicates that the quality of parental relationships has a reciprocal impact on children’s behavioral issues, with positive relationships being associated with fewer problems (66). Girls are often expected to embody the “good girl” role (67), which involves supporting the teacher and maintaining classroom order, reflecting additional societal expectations beyond academic achievement (68). The observed differences in behavior between girls and boys at school and home may be attributed to other factors. Girls are often socialized to be more empathetic, cooperative, and attentive, which influences their behavior in school and home settings. These socialization patterns reinforce their role as helpers and maintainers of order. Also; in many families, girls may be given more responsibilities related to household tasks and caregiving roles, fostering behaviors such as helpfulness and responsibility. This dynamic can shape their behavior in both school and home environments.

Moreover, the study highlighted the fact that girls in the majority of MENA countries, girls tend to experience greater feelings of loneliness and closeness to others compared to boys. Conservative cultural norms may restrict girls’ social interactions more than boys’, leading to reduced opportunities for social engagement. This restriction can contribute to feelings of loneliness as girls have fewer avenues to build and maintain friendships. Another possible explanation is that if girls’ relationships become unsatisfying, they may be more prone to experiencing loneliness and may not actively seek alternatives (69). Additionally, girls are often socialized to be more emotionally expressive and sensitive. This emotional intensity may make them more aware of and affected by feelings of loneliness, as they are more attuned to their emotional states. These findings enhance the importance of understanding and addressing the emotional well-being of adolescent girls in the MENA region. The heightened sense of loneliness and closeness to others among girls highlights potential areas for targeted interventions aimed at promoting mental health and fostering supportive social environments.

This meta-analysis has some strengths: (1) The study covers a wide range of health behaviors and across 17 countries in the MENA region, providing a comprehensive overview, (2) the use of nationally representative GSHS datasets ensures the findings are broadly applicable to each country included in the study, (3) the inclusion of the latest surveys conducted between 2007 and 2016 enhances the relevance and timeliness of the study’s findings. However, some limitations must be taken into account when interpreting the results. While the study covers a broad range of health behaviors, certain important variables or subgroups may not have been included or analyzed. The fact that the associated factors were self-reported by participants is another limitation to take into account. Also, the age groups targeted varied between countries, potentially affecting comparability across regions.

## Conclusion

This meta-analysis of adolescent populations across 17 MENA countries reveals significant gender disparities in health behaviors. The study’s comprehensive approach, utilizing recent and representative data, highlights these differences. Specifically, boys frequently exhibit higher rates of risky behaviors such as tobacco use and physical aggression, while girls generally display more helpful behaviors and heightened emotional sensitivity.

The findings underscore the need to address a wide range of unhealthy behaviors including dietary habits, hygiene practices, tobacco and drug use, physical activity, and mental health to improve overall well-being among adolescents. Preventive strategies should be region-specific, taking into account gender differences and local contexts. Understanding youth preferences for public health interventions is crucial for identifying effective measures and trends.

Addressing these gender disparities and promoting healthy behaviors is vital, particularly during the critical developmental stages of adolescence and young adulthood. Gender plays a significant role in health inequities, and reducing these disparities should be a priority in the MENA region. Additionally, interventions should incorporate curricula that promote positive daily practices to counteract unhealthy attitudes and behaviors.

## Data Availability

The raw data supporting the conclusions of this article will be made available by the authors, without undue reservation.
