# Supplementary material for: The health behaviors differences among male and female school-age adolescents in the Middle East and North Africa region countries: a meta-analysis of the Global School-based Student Health Survey data
Source: Front Public Health. 2024 Aug 26;12:1448386. doi: 10.3389/fpubh.2024.1448386 (PMC11381380; doi:10.3389/fpubh.2024.1448386)
Supplement: Supplementary file 1 [file Table_1.DOCX]

#### **Supplementary file 1.** Description of dichotomous questions^a^.

| Variable | Formulating question | Codes ^b^ |
| --- | --- | --- |
| Hunger: QN6 | Percentage of students who most of the time or always went hungry (because there was not enough food in their home during the 30 days before the survey). | 1=Yes, 2=No, blank= missing responses |
| Psychological |  |  |
| Loneliness: QN22 | Percentage of students who most of the time or always felt lonely (during the 12 months before the survey). | 1=Yes, 2=No, blank= missing responses |
| Anxiety: QN23 | Percentage of students who most of the time or always were so worried about something that they could not sleep at night (during the 12 months before the survey). | 1= Yes, 2=No, blank= missing responses |
| Suicidal ideation: QN24, QN25 | Percentage of students who seriously considered attempting suicide (during the 12 months before the survey).  Percentage of students who made a plan about how they would attempt suicide (during the 12 months before the survey).  Percentage of students who attempted suicide (one or more times during the 12 months before the survey). | 1= Yes, 2=No, blank= missing responses |
| Close friends: QN27 | Percentage of students who did not have any close friends. | 1= Yes, 2=No, blank= missing responses |
| Behavioral |  |  |
| Fruit consumption : QN7 | Percentage of students who did eat fruit 2 or more times per day (during the 30 days before the survey). | 1= Yes, 2=No, blank= missing responses |
| Vegetable consumption : QN8 | Percentage of students who did eat vegetables 3 or more times per day (during the 30 days before the survey). | 1= Yes, 2=No, blank= missing responses |
| Soft drinks : QN9 | Percentage of students who did not drink carbonated soft drinks (excluding diet soft drinks, during the 30 days before the survey). |  |
| Fast foods : QN10 | Percentage of students who did not eat food from a fast-food restaurant (during the 7 days before the survey). |  |
| Walking or biking to school : QN50 | Percentage of students who did not walk or ride a bicycle to or from school (during the 7 days before the survey) | 1= Yes, 2=No, blank= missing responses |
| Attendance of physical education :QN51 | Percentage of students who did not attend physical education classes (each week during this school year). | 1= Yes, 2=No, blank= missing responses |
| Physical inactivity : QN49, QN52 | Percentage of students who were not physically active (for at least 60 minutes per day on any day during the 7 days before the survey).  Percentage of students who spent three or more hours per day doing sitting activities (sitting and watching television, playing computer games, talking with friends when not in school or doing homework during a typical or usual day) | 1= Yes, 2=No, blank= missing responses |
| Violence and injuries |  |  |
| Physical fight : QN15 and QN16 | Percentage of students who were physically attacked (one or more times during the 12 months before the survey).  Percentage of students who were in a physical fight (one or more times during the 12 months before the survey). | 1= Yes, 2=No, blank= missing responses |
| Injuries: QN17 and QN18  QN19 | Percentage of students who were seriously injured (one or more times during the 12 months before the survey).  Percentage of students who reported that their most serious injury was a broken bone or dislocated joint (among students who were seriously injured during the 12 months before the survey).  Percentage of students who reported that their most serious injury was caused by a motor vehicle accident or being hit by a motor vehicle (among students who were seriously injured during the 12 months before the survey). | 1= Yes, 2=No, blank= missing responses |
| Tobacco use :QN28 to QN33 | QN28:Percentage of students who tried a cigarette before age 14 years (for the first time among students who ever smoked cigarettes). | 1= Yes, 2=No, blank= missing responses |
|  | QN29 : Percentage of students who currently smoked cigarettes (on at least 1 day during the 30 days before the survey). |  |
|  | QN30: Percentage of students who use any tobacco products other than cigarettes, such as Shisha, Tabac a snifer, Kala? (on at least 1 day during the 30 days before the survey). |  |
|  | QN31 : Percentage of students who tried to stop smoking cigarettes, during the past 12 months? |  |
|  | QN32: on how many days have people smoked in your presence. (on at least 1 day during the 7 days before the survey). |  |
|  | QN33 : Which of your parents or guardians use any form of tobacco? |  |
| illicit drug use : QN40 to QN43 | QN 40 : Percentage of students who used drugs before age 14 years (for the first time among students who ever used drugs). | 1= Yes, 2=No, blank= missing responses |
|  | QN41 : Percentage of students who ever used marijuana (one or more times during their life). |  |
|  | QN42 : Percentage of students who currently used marijuana (one or more times during the 30 days before the survey). |  |
|  | QN43 : Percentage of students who ever used amphetamines or methamphetamines (one or more times during their life). |  |
| School attendance : QN53 | Percentage of students who missed classes or school without permission (on one or more days during the 30 days before the survey). | 1= Yes, 2=No, blank= missing responses |
| Social |  |  |
| Peer support in school : QN54 | Percentage of students who reported that most of the students in their school were most of the time or always kind and helpful (during the 30 days before the survey). | 1= Yes, 2=No, blank= missing responses |
| Parental supervision : QN55 | Percentage of students who reported that their parents or guardians most of the time or always checked to see if their homework was done (during the 30 days before the survey). | 1= Yes, 2=No, blank= missing responses |
| Parental connectivity :QN56 | Percentage of students who reported that their parents or guardians most of the time or always understood their problems and worries (during the 30 days before the survey). | 1= Yes, 2=No, blank= missing responses |
| Parental bonding :QN57 and QN58 | QN57 : Percentage of students who reported that their parents or guardians most of the time or always really knew what they were doing with their free time (during the 30 days before the survey). | 1= Yes, 2=No, blank= missing responses |
|  | QN58 : Percentage of students who reported that their parents or guardians never or rarely went through their things without their approval (during the 30 days before the survey). |  |
| Bullying : QN20 and QN21 | QN20: Percentage of students who were bullied (on one or more days during the 30 days before the survey).  QN21 : Percentage of students who were bullied most often by being hit, kicked, pushed, shoved around, or locked indoors (among students who were bullied during the 30 days before the survey). | 1= Yes, 2=No, blank= missing responses |
| Hygiene module |  |  |
| Teeth hygiene : QN11 | Percentage of students who usually cleaned or brushed their teeth (one or more times per day during the 30 days before the survey). | 1= Yes, 2=No, blank= missing responses |
| Hands hygiene : QN12 to QN14 | QN12: Percentage of students who never or rarely washed their hands before eating (during the 30 days before the survey).  QN13: Percentage of students who never or rarely washed their hands after using the toilet or latrine (during the 30 days before the survey).  QN14 : Percentage of students who never or rarely used soap when washing their hands (during the 30 days before the survey). | 1= Yes, 2=No, blank= missing responses |

^a:^ Dichotomous questions were from the GSHS and literature.

^b^: Codes were made according GSHS Data User's Guide. Details on these tools can be accessed at http://www.who.int/chp/gshs/methodology/en/
